# Delay in sputum smear conversion and outcomes of smear-positive tuberculosis patients: a retrospective cohort study in Bafoussam, Cameroon

**DOI:** 10.1186/s12879-015-0876-1

**Published:** 2015-03-21

**Authors:** Fabrice Nembot Djouma, Michel Noubom, Jérôme Ateudjieu, Hubert Donfack

**Affiliations:** Department of Biomedical Sciences, University of Dschang, Cameroon, PO Box 067, Dschang, Cameroon; Better Access to Health Care “MASSANTE”, PO Box 33490, Yaoundé, Cameroon; Diagnostic and Treatment Centre of Baleng, Bafoussam, Cameroon; Division of Health Operations Research, Ministry of Public Health, Cameroon, PO Box 33490, Yaoundé, Cameroon

**Keywords:** Tuberculosis, Smear non-conversion, Outcome, Cameroon

## Abstract

**Background:**

In limited resource settings, sputum smear conversion at the end of the intensive phase of tuberculosis treatment is an indicator not only of patients’ response to treatment, but also of anti-tuberculosis program performance. The objective of this study was to identify factors associated to sputum smear non-conversion at the end of the intensive phase of treatment, and the effect of smear non-conversion on the outcome of smear-positive pulmonary tuberculosis patients.

**Method:**

This retrospective cohort study was carried out on data of patients treated in the Diagnostic and Treatment Centre of Baleng, West-Cameroon from 2006 to 2012. Logistic regression models were used to evaluate the association of socio-demographic and clinical factors with delay in sputum smear conversion, and the association of this delay with treatment outcomes.

**Result:**

Out of 1425 smear-positive pulmonary tuberculosis patients treated during the study period, 1286 (90.2%) were included in the analysis. Ninety four (7.3% CI: 6.0- 8.9) patients were identified as non-converted at the end of the intensive phase of treatment. Pre-treatment smears graded 2+ and 3+ were independently associated to delay in smear conversion (p < 0.01). Years of treatment ranging from 2009 to 2012 were also associated to delay in smear conversion (p < 0.02). Delay in smear conversion was significantly associated to failure [Adjusted Odd Ratio (AOR):12.4 (Confidence Interval: CI 4.0- 39.0)] and death, AOR: 3.6 (CI 1.5- 9.0).

**Conclusion:**

Heavy initial bacillary load and treatment years ranging from 2009 to2012 were associated to sputum smear non-conversion at the end of the intensive phase of TB treatment. Also, delay in smear conversion was associated to unfavorable treatment outcomes. Patients with heavy initial bacillary load should thus be closely monitored and studies done to identify reasons for the high proportion of non-conversion among patients treated between 2009 and 2012.

## Background

Tuberculosis (TB) remains one of the world’s deadliest communicable diseases [1]. In 2013, an estimated 9.0 million people developed TB and 1.5 million died from the disease [1]. The management of this disease is a great challenge in developing countries because resources are limited, health systems are weak and the rate of HIV infection is high.

According to the WHO, all TB patients should be monitored during anti-tuberculosis treatment to assess their response to therapy [2]. The monitoring basically concerns body weight and sputum smear examination which should be done, among others, at the end of the intensive phase of treatment [2]. Despite the low positive predictive value of sputum smear examination during treatment, it has been documented to be well correlated to smear culture [3] and the proportion of smear-positive patients with sputum smear conversion at the end of the intensive phase is an indicator of TB program performance [3].

Non-conversion of sputum smear at the end of the intensive phase of treatment has been documented to be associated with unfavorable outcomes, more specifically with default and failure [4-8]. Therefore, the knowledge of associated risk factors to delay in sputum smear conversion at the end of the initiation phase of anti-tuberculosis treatment is necessary for care providers to prevent unfavorable outcomes. This study, done in a major Diagnostic and Treatment Centre (DTC) in Bafoussam, West-Cameroon had as objective to identify the risk factors of sputum smear non-conversion at the end of the intensive phase of treatment, and the effects of smear non-conversion on the outcome of SPPTB patients.

## Methods

### Study design

**Figure 1:**
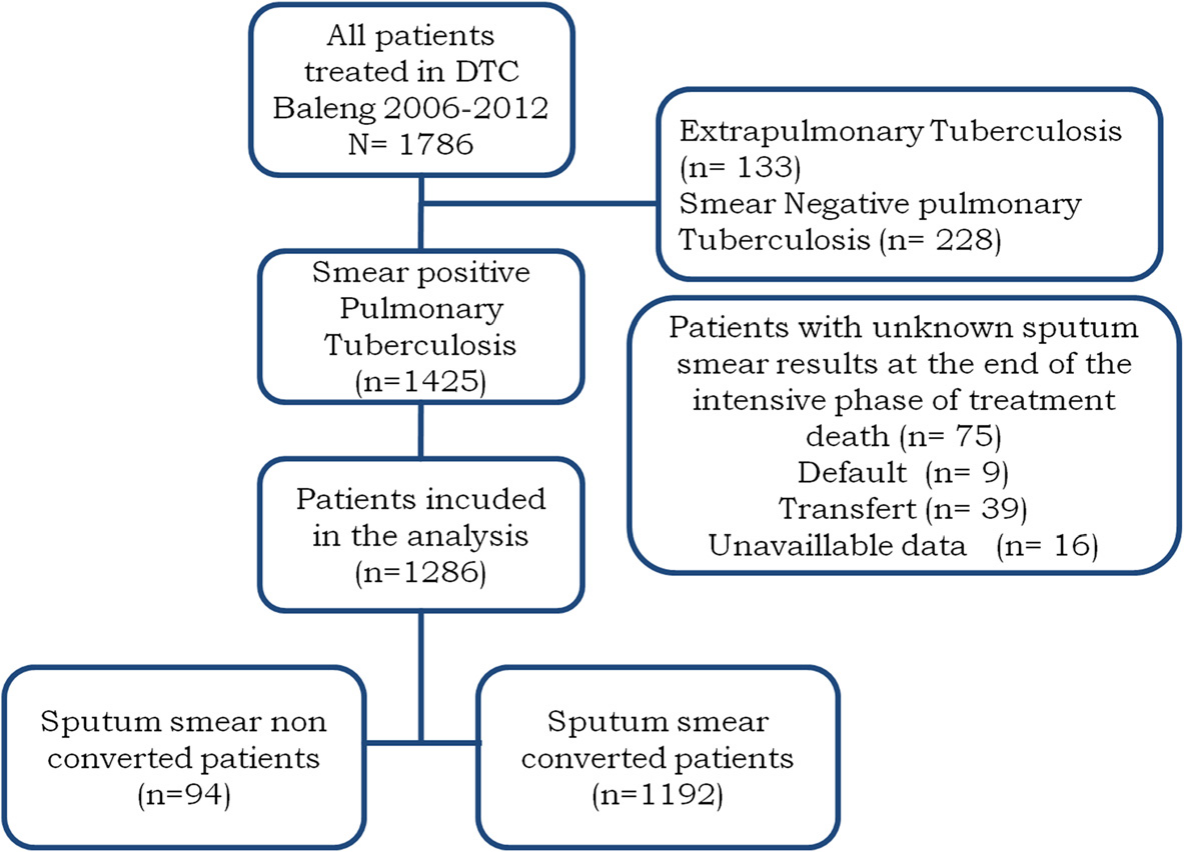
**Algorithm of repartition of Tuberculosis patients treated in DTC of Baleng from 2006 to 2012.**

It was a retrospective cohort study based on documentary review of smear-positive pulmonary TB patients registered at the DTC of Baleng, from 2006 to 2012. The main factor of interest was the non-conversion of sputum smear at the end of the intensive phase of anti-tuberculosis treatment and the effects were the different outcomes of patients at the end of treatment. Patients included in the analysis were those who had completed at least the intensive phase of anti-tuberculosis treatment (see Figure 1). Patients with unknown data for the main variables of interest were excluded from the study.

### Study site

The Diagnostic and Treatment Centre of Baleng is the largest TB treatment centre of the Cameroonian West Region. It is a public centre that covers a population of about 20000 persons. The centre also receives patients from other parts of the country, especially the West, Littoral and Centre Regions [9]. TB case management is done there according to national guidelines which have been conceived from international guidelines [4].

### Tuberculosis control in Cameroon

In Cameroon, TB diagnosis is firstly based on clinical suspicion. Suspected cases are therefore referred to one of the 238 DTCs that cover the whole country. In these TB DTCs, patients are first of all classified according to the anatomical site of TB. In this way, pulmonary and extra-pulmonary TB are distinguished. Pulmonary TB cases are subsequently classified according to bacteriological results. Bacteriology refers to the smear status of cases. Smear examination is done through microscopic observation of *M. tuberculosis* after staining using Ziehl Neelsen’s technique. After this, pulmonary TB cases are classified into smear-negative pulmonary TB (smear contains no AFB in 100 fields) and smear-positive pulmonary TB (SPPTB) groups. Smear grading of SPPTB cases is as follows: 1+ (10–99 acid-fast bacilli (AFB) in 100 fields), 2+ (1–9 AFB/field in at least 50 fields), and 3+ (>10 AFB/field in at least 20 fields).

Whatever the clinical form of TB, all patients should undergo two phases of treatment: intensive/initiation phase and continuation phase. The length of the intensive phase depends on patients’ previous history of anti-tuberculosis treatment (2 months for new patients and 3 months for previously treated patients).

In Cameroon, the implementation of Direct Observed Treatment (DOT) recommended by the WHO is too limited because DTCs are few (1 DTC for about 90000 habitants) and patients bear the indirect cost of treatment. The national program recommends to provide drugs to patients on a weekly basis during the intensive phase and on a monthly basis during the continuation phase of the treatment. Treatment consists of a two month intensive phase of daily rifampicin (R), isoniazid (H), pyrazinamide (Z) and ethambutol (E), followed by a four month continuation phase of daily R and H on outpatient basis. However, a very limited number of patients are hospitalized during the intensive phase of treatment.

At the end of the intensive phase of treatment, smears examination should be done for each SPPTB case. Depending on the results, patients can be classified into two groups: (1) Sputum smear conversion at the end of the intensive phase of treatment and (2) Sputum smear non-conversion at the end of the intensive phase of treatment (patients with persistently positive smears). Also, smear examination can be done during treatment to assess response to treatment.

At the end of follow-up, patients were classified in several groups, according to outcomes of treatment and drug-susceptible TB [1].

1. Cured: A patient who was initially sputum smear-positive but was smear-negative in the last month of treatment, and on at least one previous occasion
2. Died: A patient who died from any cause during treatment
3. Failed: A patient who was initially sputum smear-positive and remained positive at month 5 or later during treatment
4. Defaulted: A patient whose treatment was interrupted for two consecutive months or more.
5. Transfer: A patient who was transferred to another DTC during TB treatment.

### Data analysis

Data were collected from patients’ registers at DTC Baleng from 2006 to 2012. The main variables collected were socio-demographic and clinical characteristics of patients, clinical presentation of TB, results of sputum smear examination prior to and during anti-tuberculosis treatment and the patients’ outcomes at the end of follow-up.

Statistics were analyzed with Epi info software version 3.5.4 from the Center for Disease Control and Prevention (CDC). Chi square or Fisher’s exact tests were used to compare proportions. A backward logistic regression was used to identify the independent risk factors for bacteriological non-conversion at the end of the intensive phase of treatment. The same logistic regression method was used to assess the effects of sputum smear non-conversion, at the end of the intensive phase of treatment, on patients’ outcomes. A p-value <0.05 was used to characterize significant results.

As data were collected in patients’ registers, no informed consent was necessary. The study was approved by the administrative authorities of the Diagnostic and Treatment Centre of Baleng and the Mifi Health Committee.

## Results

Between 2006 and 2012, 1786 TB patients presented to DTC Baleng. Among them, 133 (7.4%) were extra-pulmonary cases and 228 (12.8%) were smear-negative pulmonary TB cases. Out of 1425 smear-positive pulmonary TB cases, 1286 (90.2%) were included in the analysis.

The mean age of patients included in the analysis was 36.7 (SD: 14.7); 772 (60% CI: 57.3- 62.7) patients were male while 514 (40%) were female. One hundred and five (8.4%) patients had previous history of TB treatment. Results of pre-treatment smear examination were as follows: 1+ grade for 182 (14.2%) patients, 2+ for 589 (45.8%) patients and 3+ for 515 (40.0%) patients. Among patients whose HIV status was available (83.4%), 238 (22.1%) were HIV positive while 839 (77.8%) were HIV negative; 42.0% of HIV positive patients were under antiretroviral treatment. Ninety four (7.3% CI: 6.0- 8.9) patients had non-converted sputum smear at the end of the intensive phase of treatment whereas 1192 (92.7 CI: 91.1- 94.0) patients had converted sputum smear at the same time; these details are presented in Figure 1.

Table 1 compares the socio-demographic and clinical characteristics of patients with and without persistently positive sputum smear at the end of the intensive phase of anti-tuberculosis treatment. This univariate analysis showed that 2+ (OR = 7.1 CI: 1.7- 29.5) and 3+ (OR = 9.5 CI: 2.2- 39.3) pre-treatment smear grades, and years of consultation from 2009 to 2012 (p < 0.01) were significantly associated to sputum smear non-conversion at the end of the intensive phase of treatment. Multivariate logistic regression analysis presented in Table 2 indicated that all significant variables from the univariate analysis were independently associated to delayed smear conversion at the end of the intensive phase of treatment. Moreover, having a previous history of tuberculosis treatment was not associated to persistently positive sputum smear at the end of the intensive phase (p = 0.5).

**Table 1:** **Characteristics of sputum smear-positive tuberculosis patients, comparing the end of intensive treatment phase non-converters (delayed converters) with converters in CDT of Baleng, 2006–2012**

**Characteristics**	**Delayed converters**		**Converters**		**Crude odd ratio (95% confidence interval)**	**p value**
	**(n = 94)**	**n (%)**	**(n = 1192)**	**n (%)**		
Age 33 years old	55 (58.5)		582 (48.8)		1.5 (0.9- 2.3)	0.04
Male sex	62 (66.0)		710 (59.6)		1.3 (0.8- 2.0)	0.22
**Years**						
2006	7 (7.4)		208 (17.5)		Reference	-
2007	1 (1.1)		147 (12.3)		0.2 (0.02- 1.7)	0.14
2008	6 (6.4)		180 (15.1)		0.9 (0.3- 3.0)	0.9
2009	26 (27.7)		178 (14.9)		4.3 (1.8- 10.2)	<0.01
2010	15 (16.0)		153 (12.8)		2.9 (1.2- 7.3)	<0.01
2011	20 (21.3)		211 (17.7)		2.8 (1.2- 6.8)	0.02
2012	19 (20.2)		114 (9.6)		4.9 (2.0- 12.1)	<0.01
Positive HIV status	20 (23.5)		218 (22.0)		1.1(0.6- 1.8)	0.74
ART* treatment	7 (38.9)		74 (42.3)		0.9 (0.3- 2.3)	0.78
Previous tuberculosis treatment history	8 (8.8)		97 (8.4)		1.1 (0.5- 2.2)	0.50
**Initial smear grading**						
1+	2 (2.1)		180 (15.1)		Reference	-
2+	43 (45.7)		546 (45.8)		7.1 (1.7- 29.5)	<0.01
3+	49 (52.1)		466 (39.1)		9.5 (2.3- 39.3)	<0.01

*Antiretroviral treatment.

**Table 2:** **Odds Ratio and p-value of associated risk factors of sputum smear non conversion at the end of intensive phase of treatment determined from multivariable logistic regression model**

**Characteristic**	**Adjusted odd ratio (95% confidence interval)**	**p value**
Age 33 years old	1.5 (0.9- 2.4)	0.07
Male sex	1.2 (0.7- 1.9)	0.55
**Years of consultation**		
2006	Reference	-
2009	4.4 (1.8- 10.4)	<0.01
2010	3.1 (1.2- 8.0)	0.02
2011	3.5 (1.4- 8.5)	<0.01
2012	5.4 (2.2- 13.4)	<0.01
**Initial smear grading**		
1+	Reference	
2+	9.9 (2.4- 42.4)	<0.01
3+	10.6 (2.5- 44.5)	<0.01

At the end of individual follow-up, 1119 (87.0% CI: 85.0- 88.8) patients were cured; 43 (3.3% CI: 2.4- 4.5) died; 17 (1.3% CI: 0.8- 2.2) failed, 52 (4.0% CI: 3.1- 5.3) defaulted and 55 (4.3% CI: 3.3- 5.6) were transferred to other DTCs.

As shown in Table 3, univariate analysis associated non-conversion of sputum smear at the end of the intensive phase of treatment with failure (OR = 7.9 CI: 2.8- 22.1) and death (OR = 3.3 CI: 1.7- 7.4) of SPPTB patients. On the contrary, delay in sputum smear conversion at the end of the intensive phase of anti-tuberculosis treatment was not significantly associated to default (p = 0.56) and transfer (p = 0.29). After adjustment by multivariate logistic regression analysis (Tables 4 and 5), persistently positive sputum smear at the end of the intensive phase of anti-tuberculosis treatment remained associated to failure (AOR = 11.2 CI: 3.5- 35.2) and death (1OR = 3.7 CI: 1.5- 9.0).

**Table 3:** Univariable analysis of association between sputum smear non conversion and final outcomes of smear positive pulmonary tuberculosis in CDT* of Baleng, 2006- 2012

**Characteristics**	**Delayed converters**		**Converters**		**Crude odd ratio (95% confidence interval)**	**p value**
	**(n = 94)**	**n (%)**	**(n = 1192)**	**n (%)**		
Cured	72 (76.6)		1047 (87.8)		Reference	-
Died	8 (8.5)		35 (2.9)		3.3 (1.5- 7.4)	<0.01
Failed	6 (6.3)		11 (1.0)		7.9 (2.8- 22.1)	<0.01
Defaulted	3 (3.3)		49 (4.1)		0.9 (0.3- 2.9)	0.56
Transferred	5 (5.3)		50 (4.2)		1.4 (0.6- 3.8)	0.29

*Diagnostic and Treatment Centre.

**Table 4:** Multivariable logistic regression analysis accessing the association between sputum smear non conversion at the end of intensive phase of TB treatment and treatment failure in DTC* of Baleng, 2006–2012

**Characteristic**	**Adjusted odd ratio (95% confidence interval)**	**p value**
Male sex	1.9 (0.6- 6.2)	0.30
Age˃ 33	0.6 (0.2- 1.6)	0.29
Previous tuberculosis treatment history	11.2 (3.5- 35.3)	<0.01
Non conversion of sputum smear at the end of intensive phase	12.4 (4.0- 39.0)	<0.01

*Diagnostic and Treatment Centre.

**Table 5:** Multivariable logistic regression accessing the association between sputum smear non conversion at the end of intensive phase of TB treatment and patients’ death in DTC* of Baleng, 2006–2012

**Characteristic**	**Adjusted OR (95% confidence interval)**	**p value**
Male sex	1.2 (0.6- 2.7)	0.54
Age˃ 33	1.4 (0.6- 2.9)	0.41
Positive HIV status	6.8 (3.3- 14.0)	< 0.01
Non conversion of sputum smear at the end of intensive phase	3.6 (1.5- 9.0)	< 0.01

*Diagnostic and Treatment Centre.

## Discussion

The first objective of this study was to identify the risk factors of sputum smear non-conversion at the end of the intensive phase of treatment. Analysis done on socio-demographic and clinical data of SPPTB treated in the DTC of Baleng between 2006 and 2012 showed that high pre-treatment smear grades (2+ and 3+) and treatment years between 2009 and 2012 were significantly and independently associated to delay in sputum smear conversion at the end of the intensive phase of treatment.

The limitation to this study is that analysis was based on data collected from patients’ registers; therefore potential risk factors not found in the registers were not assessed.

The proportion of smear-positive patients with sputum smear non-conversion at the end of the intensive phase is an indicator of TB program performance [3]. During the study period, 7.3% of SPPTB patients had delayed sputum smear conversion at the end of the initial phase of treatment. Studies done in many settings have shown that proportions of sputum smear non-conversion at the end of the intensive phase of TB treatment range from 5% to 32% [8,10-13]. Many reasons can explain the non-conversion of sputum smear at the end of the intensive phase of TB treatment. First of all, non-viable bacteria remain visible by microscopy. Ideally, culture is the best method to evaluate the viability of *M. Tuberculosis* [14]. Unfortunately, this method can’t be used in field conditions, especially in limited settings such as in developing countries. However, a study has shown a good correlation between culture and sputum acid-fast bacilli smear [5]. Therefore, the use of microscopic observation to evaluate the non-conversion rate at the end of the intensive phase of treatment can be acceptable. Other potential explanations for non-conversion of sputum smear at the end of the intensive phase of TB treatment are: poor supervision of the initial phase of therapy, poor patient adherence, poor quality of anti-TB drugs, doses of anti-TB drugs below the recommended range, co-morbid conditions, drug-resistant *M. tuberculosis* that is not responding to first-line treatment and heavy initial bacillary load [4]. Heavy initial bacillary load has been documented (as in our study) as an important risk factor of delay in sputum smear conversion at the end of intensive phase of TB treatment [6,7,15-17]. So, to avoid non-conversion at the end of the initial phase of TB treatment, patients should be diagnosed early and treated. And moreover, further studies are needed to identify risk factors of diagnostic and treatment delay among suspected TB patients and their effects on initial bacillary load.

Previous studies have identified older age [9,18] and previous TB treatment history [7,18] as significant risk factors of sputum smear non-conversion at the end of the intensive treatment phase. In this study however, these associations were not significant. This may be due to sample fluctuation. In contrary to older age and previous TB treatment history, years of treatment ranging from 2009 to 2012 were significantly associated to sputum smear non- conversion at the end of the intensive phase of treatment. This can be the consequence of augmentation of drug-resistant *M. tuberculosis* that is not responding to first-line treatment or a change in TB case management compared to previous years. Further studies are thus necessary to verify these hypotheses.

The second objective of this study was to evaluate the association between sputum smear non-conversion at the end of the intensive phase of treatment and the final outcomes of patients. Results have shown that sputum smear non-conversion was independently associated to failure and death. Many previous studies have established the association between sputum smears non-conversion and unfavorable final treatment outcomes, especially failure [6,7,19-21]. Failure is generally the main outcome identified individually during evaluation of sputum smear non-conversion effects on final TB cases outcomes. This can be due to the small number of deaths, defaults and transfers among cohorts of TB patients in general and particularly among those who complete the intensive phase of treatment [22,23]. However, this study has shown that smear non-conversion is associated to TB patients’ death. This emphasizes the need to reduce the proportion of sputum smear non-conversion, thereby preventing unfavorable outcomes.

## Conclusion

Heavy initial bacillary load and treatment years ranging from 2009 to 2012 were associated sputum smear non-conversion at the end of the intensive phase of treatment among smear-positive pulmonary TB patients who presented in the Baleng Diagnostic and Treatment Centre from 2006 to 2012. Delay in smear conversion was associated to failure and death. Patients with heavy initial bacillary load should be closely monitored to prevent sputum smear non-conversion at the end of the intensive phase of treatment and unfavorable outcomes. Moreover, further studies are needed to identify risk factors of heavy initial bacillary load among smear-positive pulmonary tuberculosis patients.

## Abbreviations

TB: Tuberculosis
SPPTB: Smear-Positive Pulmonary tuberculosis

## References

[1] World Health Organisation (WHO). Global tuberculosis report; 2014. http://apps.who.int/iris/bitstream/10665/91355/1/9789241564656_eng.pdf. Accessed 25 Dec 2014.

[2] World Health Organisation (WHO) (2010). Treatment of tuberculosis: guidelines.

[3] Hobby GI, Holman AP, Iseman MD, Jones JM (1973). Enumeration of tubercle bacilli in sputum of patients with pulmonary tuberculosis. Antimicrob Agents Chemother.

[4] Kuaban C, Bame R, Mouangue L, Djella S, Yomgni C (2009). Non Conversion Of Sputum Smears In New Smear Positive Pulmonary Tuberculosis Patients In Yaoundé, Cameroon. East Afr Med J.

[5] Dominguez-Castellano A, Muniain MA, Rodriguez-Bano J, Garcia M, Rios MJ, Galvez J (2003). Factors associated with time to sputum smear conversion in active pulmonary tuberculosis. Int J Tuberc Lung Dis.

[6] Pefura-Yone EW, Kengne AP, Kuaban C (2014). Non-conversion of sputum culture among patients with smear positive pulmonary tuberculosis in Cameroon: a prospective cohort study. BMC Infect Dis.

[7] Ukwaja KN, Oshi DC, Oshi SN, Alobu I (2014). Profile and treatment outcome of smear positive TB patients who failed to smear convert after 2 months of treatment in Nigeria. Trans R Soc Trop Med Hyg.

[8] Salaniponi FML, Christensen JJ, Gausi F, Kwanjana JJ, Harries AD (1999). Etat de la bacilloscopie à deux mois et résultat final subséquent du traitement chez les nouveaux patients atteints d’une tuberculose pulmonaire à bacilloscopie positive. Int J Tuberc Lung Dis.

[9] Noubom M, Djouma NF, Donfack H, Kouomboua NPK, Tchasse T (2013). Caracterisitiques des patients tuberculeux à l’ouest cameroun: 2000–2009. Pan Afr Med J.

[10] Jayakody W, Harries AD, Malhotra S (2013). Characteristics and outcomes of tuberculosis patients who fail to smear convert at two months in Sri Lanka. Public Health Action.

[11] Singla R, Osman MM, Khan N (2003). Factors predicting sputum smear positivity among pulmonary tuberculosis patients 2 months after treatment. Int J Tuberc Lung Dis.

[12] Parikh R, Nataraj G, Kanade S (2012). Time to sputum conversion in smear positive pulmonary TB patients on Category 1 DOTS and factors delaying it. J Assoc Physicians India.

[13] Kuyp VF, Mahan CS (2012). Prolonged positivity of sputum smears with negative cultures during treatment for pulmonary tuberculosis. Int J Tuberc Lung Dis.

[14] Bouti K, Aharmim M, Marc K, Soualhi M, Zahraoui R, Benamor J (2013). Factors influencing sputum conversion among smear-positive pulmonary tuberculosis patients in morocco. ISRN Pulmonology.

[15] Tiwari S, Kumar A, Kapoor SK (2012). Relationship between sputum smear grading and smear conversion rate and treatment outcome in the patients of pulmonary tuberculosis undergoing dots: a prospective cohort study. Indian J Tuberc.

[16] Rekha VVB, Balasubramanian R, Swaminathan S, Rahman RRF, Sundaram V, Thyagarajan K (2007). Sputum conversion at the end of intensive phase of Category-1regimen in the treatment of pulmonary tuberculosis patients with diabetes mellitus or HIV Infection: An analysis of risk factors. Indian J Med Res.

[17] Fanai S, Viney K, Tarivonda L, Roseveare C, Tagaro M, Marais BJ (2014). Profile of tuberculosis patients with delayed sputum smear conversion in the Pacific island of Vanuatu. Public Health Action.

[18] Lee J, Lee BJ, Yoon HI, Lee C-T, Lee JH (2012). Influence of previous tuberculosis treatment history on acid-fast bacilli smear and culture conversion. Int J Tuberc Lung Dis.

[19] Feng-Zeng Z, Levy MH, Sumin W (1997). Sputum microscopy results at two and three months predict outcome of tuberculosis treatment. Int J Tuberc Lung Dis.

[20] Rieder HL (1996). Sputum smear conversion during directly observed treatment for tuberculosis. Tuber Lung Dis.

[21] Lienhardt C, Manneh K, Bouchier V, Lahai G, Milligan PJM, McAdam KPWJ (1998). Factors determining the outcome of treatment of adult smear-positive tuberculosis cases in the Gambia. Int J Tuberc Lung Dis.

[22] Schmaltz CAS, Santoro-Lopes G, Lourenc MC, Morgado MG, Velasque LS (2012). Factors Impacting Early Mortality in Tuberculosis/HIV Patients: Differences between Subjects Naïve to and Previously Started on HAART. PLoS One.

[23] Dodor EA (2004). Tuberculosis treatment default at the Communicable Diseases Unit of Effia-Nkwanta Regional Hospital: a 2 year experience. Int J Tuberc Lung Dis.

